# Nitrogen-doped carbon dots enhanced seedling growth and salt tolerance with distinct requirements of excitation light†

**DOI:** 10.1039/d3ra01514a

**Published:** 2023-04-18

**Authors:** Xiuli Jing, Yankai Liu, Xuzhe Liu, Xiao-Fei Wang, Chunxiang You, Dayong Chang, Shuai Zhang

**Affiliations:** a State Key Laboratory of Crop Biology, Shandong Green Fertilizer Technology Innovation Center, Apple Technology Innovation Center of Shandong Province, College of Horticulture Science and Engineering, Shandong Agricultural University Taian Shandong China; b Key Laboratory of Agricultural Film Application of Ministry of Agriculture and Rural Affairs, College of Chemistry and Material Science, Shandong Agricultural University Taian Shandong China hyzs@sdau.edu.cn; c Yantai Goodly Biological Technology Co., Ltd. Yantai Shandong China ytgoodly@163.com

## Abstract

Numerous nanomaterials with optical properties have demonstrated excellent capacities to enhance plant growth and stress tolerance. However, the corresponding mechanisms have only been partially characterized, especially the excitation-light dependencies of different actions. Here, nitrogen-doped carbon dots (N-CDs) were developed to explore the excitation-light dependence in N-CD-induced growth enhancement and salt tolerance. Compared to the control, N-CDs induced significant enhancements in *Arabidopsis thaliana* growth under excitation light, including fresh/dry weight of shoot (21.07% and 16.87%), chlorophyll content (9.17%), soluble sugar content (23.41%), leaf area (28.68%), total root length (34.07%) and root tip number (46.69%). In the absence of excitation light, N-CD-treated seedlings exhibited little differences in these parameters, except the enhancements in root length (24.51%) and root tip number (10.24%). On the other hand, N-CD-treatment could improve seedling salt tolerance with or without excitation light. Under salt stress (150 mM NaCl), in the presence of excitation light, the N-CDs treatment significantly increased shoot/root fresh weight and chlorophyll content by 43.29%, 50.66% and 22.59%, and reduced malondialdehyde (MDA) content and relative conductivity by 17.59% and 32.58% compared to the control group. In the absence of excitation light, significant enhancements in shoot/root fresh weight (34.22%, 32.60%) and chlorophyll content (10.45%), and obvious decreases in MDA content (28.84%) and relative conductivity (16.13%) were also found. These results indicated that N-CDs only induced growth enhancement under excitation light, but they improved salt tolerance with and without excitation light, suggesting that the two effects occurred *via* distinct signaling pathways. This study revealed the excitation-light dependencies of nanomaterial-involved agriculture applications, providing insight into designing more efficient nanomaterials in the future.

## Abstract 2

The nitrogen-doped carbon dots (N-CDs) only induced growth enhancement under excitation light, but they improved salt tolerance with and without excitation light.

## Graphical abstract

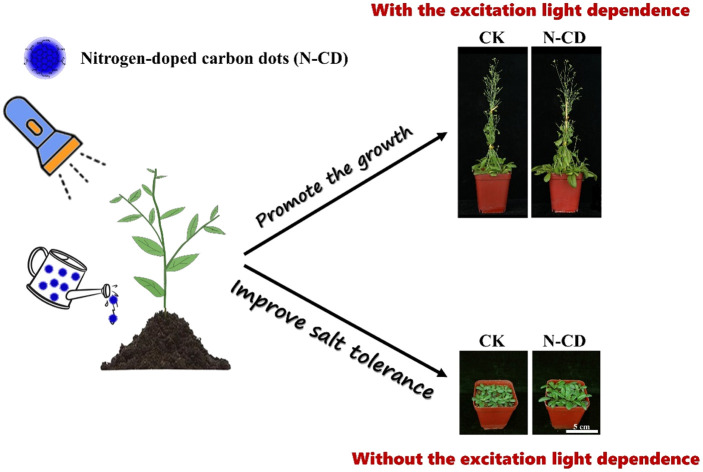

## Introduction

As the cornerstone of modern society, agriculture and the food security it provides are critical to sustainable human development.^1,2^ Although the green revolution has enabled crops to increase yields year-over-year, the increasing demand on agricultural production due to the growing population is flattening enhancement gains.^3,4^ Furthermore, soil degradation and climate change have exerted severe stresses on modern crops, threatening global food safety.^5^ It was found that approximately 50% of crop yield losses were caused by biotic/abiotic stresses, such as increased soil salinity, water deficiency, crop pests, land desertification, *etc.*^6–8^ To address these problems, several strategies have allowed modest increases in global crop yields, primarily through improving the cultivation environment,^9–11^ innovations in agrochemicals,^12,13^ and the exploitation of genetic engineering techniques.^14–17^ Despite the great achievements in growth enhancement and stress resistance achieved through these strategies, several questions regarding inefficiency, high pollution, and social controversies remain. Therefore, efficient strategies to enhance both growth and stress tolerance are still research hotspots in the agriculture field.

Recently, researches in agriculture nanotechnology, referred to as “plant nanoscience”, have produced promising results in recent years, demonstrating its potential as a tool for improving plant growth and stress tolerance.^18,19^ Nanomaterials (<100 nm in size) have excellent optical properties, environmental friendliness, high biocompatibility, and high cost performance efficiency, and they can easily enter plant cells where they regulate plant physiological activities.^20^ The effects of nanomaterials on plant growth and stress tolerance have been widely reported.^19^ Among the examples, single-walled carbon nanotubes (SWNTs) have been shown to localize within chloroplasts and promote photosynthetic activity.^21^ It was also reported that silica nanoparticles (NPs) not only improved seedling photosynthesis in wheat and lupin, but also protected rice against abiotic/biotic stresses.^22,23^ Similarly, Wu *et al.* demonstrated that cerium oxide (CeO_2_) NPs with a low Ce^3+^/Ce^4+^ ratio protected seedling photosynthesis by scavenging reactive oxygen species (ROS) induced by abiotic stresses.^24^ Rossi *et al.* showed that CeO_2_ NPs altered Na^+^ fluxes and reduced Na^+^ accumulation in roots under salt stress, resulting in a better performance of Brassica.^25^ Collectively, numerous nanomaterials have shown to have the capacity to promote seedling growth and stress tolerance.

As a promising carbon-based nanomaterial family, carbon dots (CDs) have shown excellent biocompatibility and optical performance, and in the last decade have been shown to have a wide variety of applications in the nanoagriculture field,^20^ exhibiting beneficial effects on seedling germination,^26–28^ photosynthesis enhancement,^29–40^ and stress resistance.^41–50^ For instance, degradable CDs,^39^ far-red CDs,^51^ element-doped CDs,^32,52–54^ glucose-functionalized CDs,^55^ and spirulina-based CDs^56^ have all demonstrated outstanding abilities to enhance photosynthesis and plant growth. Several mechanisms of how CDs affect photosynthesis have been proposed. Electron/energy transfer from CDs to chloroplasts^40,57,58^ and light conversion capacity^59,60^ have been suggested to contribute to these effects, although no conclusive evidence has been obtained. Furthermore, remarkable progress has been made in using CDs for stress tolerance enhancement, such as carbon dots (CDs) with different oxygen contents,^30^ salvia miltiorrhiza-derived CDs^48,49^ and nitrogen/sulfur co-doped CDs.^61^ In enhancing stress tolerance, CDs have been shown to reduce stress-induced oxidative damage, probably due to their ROS scavenging capacities^49,62^ and ROS-independent Ca^2+^ mobilization.^48^

Despite these great achievements, the underlying mechanisms have not been fully characterized, especially the excitation-light dependencies. As optical excitation nanomaterials, the roles of excitation light in these two CD-induced effects (improved growth and stress tolerance) have not been systemically explored. Recently, xenon lamps mimicking natural sunlight were employed,^37,51,63,64^ and in other cases no information on cultivation light was provided,^32,35,43,54,60^ leading to authors neglecting the effect of excitation light dependence. Therefore, this work explored the mechanisms underlying the effects of nitrogen-doped carbon dots (N-CDs) in enhancing seedling growth and salt tolerance. Specifically, the excitation light requirements of the two actions were shown to differ, indicating that N-CDs promoting plant growth and stress resistance had distinct mechanisms of action. Taken together, this work provides new insight into the application potential of CDs in agriculture.

## Experimental

### N-CDs synthesis

N-CDs were synthesized *via* hydrothermal treatment of diethylenetriamine and citric acid.^65^ In detail, 0.5 g citric acid and 280 μL diethylenetriamine were dissolved in 10 mL distilled water in a 100 mL Teflon-lined stainless steel autoclave. The mixture was heated at 180 °C for 4 h to obtain crude N-CD solutions. The N-CDs were further purified by filtration, freeze-drying, and acetone precipitation to finally obtain yellow-brown powders. To determine the effect of nitrogen doping on the optical band gap of N-CDs, undoped CDs were also prepared through the similar procedure, in which no diethylenetriamine was used.

### N-CDs characterization

The UV-vis absorption spectra were measured using a Shimadzu UV3600 UV-vis absorption spectrophotometer and the corresponding optical band gaps (*E*_g_) of CDs were estimated through the Tauc plot [the curve of (*αhν*)^2^*versus hν* converted from the UV-vis spectrum].^66^ The photoluminescence spectra were characterized using a fluorescence spectrophotometer (LUMINA, Thermo Scientific). The morphology of the N-CDs was observed by transmission electron microscope (TEM, FEI Tecnai G2 F20) and atomic force microscope (AFM, Bruker Dimension Icon), and the particle size was calculated using the Nano Measurer software. The XRD spectra were measured by Rigaku SmartLab SE (2*θ* = 10°–90°). The structure and composition of the N-CDs were examined by X-ray photoelectron spectrometer (XPS, Thermo Scientific ESCALAB 250Xi) and Fourier transform infrared spectrophotometer (FT-IR). The zeta potentials of the N-CDs were determined by nanoparticle size analyzer (Zetasizer nano ZS90, Malvern) under different pH conditions.

### Plant materials and growth conditions

#### Arabidopsis thaliana

The *Arabidopsis* (wild-type Col-0) seeds were sterilized with 75% ethanol and 2% sodium hypochlorite and sown in 1/2 MS medium.^10^ After vernalization (4 °C, darkness) for 72 h, they were transferred into a light incubator (22 °C, light/dark 16 h/8 h). After 3 days, seedlings with uniform growth were selected and used in different experiments. (1) Some seedlings were transplanted into soil environments (100% vermiculite) and acclimatized in the greenhouse for 10 days under normal light illumination (20.4267 μmol m^−2^ s^−1^). After acclimation, the seedlings were irrigated with or without N-CDs (300 mg L^−1^) and with or without excitation light (*λ*_max_ = 365 nm, 0.8325 μmol m^−2^ s^−1^), respectively. The seedlings were photographed and phenotypes were recorded at 20 days, at which point the bolting stems were cut to allow better observation of the growth pattern of the rosette leaves. The fresh and dry weights, chlorophyll contents, soluble sugar contents, root and leaf parameters, and other related indices were measured. (2) Seedlings were transplanted into soil and acclimatized for 10 days under normal light illumination. Then the seedlings were irrigated with 150 mM NaCl solution with or without N-CDs (300 mg L^−1^) and with or without excitation light (*λ*_max_ = 365 nm, 0.8325 μmol m^−2^ s^−1^). After 14 days of cultivation the relevant indices were measured, including fresh weight, chlorophyll content, MDA content, relative conductivity, and *F*_v_/*F*_m_ value. (3) Some seedlings were transferred directly to 1/2 MS medium containing 0/50/100/150 mM NaCl with or without the addition of N-CDs (300 mg L^−1^) and with or without excitation light (*λ*_max_ = 365 nm, 0.8325 μmol m^−2^ s^−1^), respectively. After 14 days of cultivation, the fresh weight, chlorophyll content, and other indices were measured.

#### Malus hupehensis

The seeds of *M. hupehensis* were soaked in water for 2 days and laminated for 1–2 months at 4 °C under dark conditions. The seeds were then sown in soil (vermiculite : soil substrate = 1 : 2) and cultured in a greenhouse. Seedlings with two true leaves and uniform growth were transplanted into Hoagland nutrient solution (Table S1†) for hydroponic culture. After 2 days of acclimatization, they were transferred to Hoagland nutrient solution with or without N-CDs (300 mg L^−1^) in the presence of excitation light (*λ*_max_ = 365 nm, 0.8325 μmol m^−2^ s^−1^) for 30 days. Then the relevant indexes, such as fresh weight, chlorophyll content, soluble sugar content, root development, and leaf parameters, were examined.

The light source and its light wavelength (in nm) & spectra used in this study were in Fig. S1 and Table S2.†

### Uptake and distribution of N-CDs in *Arabidopsis*

After 20 days of culturing in N-CD solutions (300 mg L^−1^), images of the control and N-CDs treated groups were taken in dark room under excitation light (*λ*_max_ = 365 nm, 0.8325 μmol m^−2^ s^−1^) irradiation to observe the distribution of N-CDs in the whole *Arabidopsis* plant. Further, the fluorescence of different tissues such as roots, leaves, and fruit pods in *Arabidopsis* seedlings was observed by high-resolution confocal laser microscopy (LSM 880) to confirm the distribution of N-CDs in plants (408 nm excitation, 430–500 nm collection).

### Measurement of plant physiology and growth

#### Plant weight

Several plant seedlings were randomly selected from each treatment, then weighed for fresh weight. Subsequently, they were killed at 105 °C for 30 minutes and dried at 80 °C to a constant weight to obtain the dry weight.

#### Stem length

Images of entire plants were taken against a black background and then the stem lengths were measured using the ImageJ (v1.52p) software.

#### Root system shape parameters

Pictures of root scans were analyzed using the WinPHIZO Pro 2019 software to obtain the total root length and root tips.

#### Leaf parameters

The leaf area and leaf number of plant leaves were measured using the ImageJ (v1.52p) software.

#### Elemental content

The elemental content was measured based on previous experimental methods with appropriate modifications.^67^ Completely dried plant materials from different treatments were ground into powder. 0.5 g of each sample was placed into a 50 mL conical flask and 5 mL concentrated sulfuric acid was added, then the mixture was shaken well and left overnight. The samples were digested by heating to 340 °C on an electric hot plate. When a few bubbles appeared in the liquid and white haze appeared in the triangular flask, 30% hydrogen peroxide was added dropwise until the solution became clear. The boiling product was diluted to 50 mL to obtain the final elemental solution which would be measured. After filtering through a 0.22 μm membrane, the elemental content was determined by ICP-AES.

#### Soluble sugar content

The soluble sugar content was determined using the phenol method.^68^ Samples were extracted in boiling water for 30 min. After cooling, the water with sample extract was centrifuged at 12 000 rpm for 10 min and the supernatant was placed into a new 15 mL centrifuge tube. The process was repeated twice to obtain the extract solution. Subsequently, 0.5 mL the extract solution, 1.5 mL distilled water, 1 mL 0.09 g mL^−1^ phenol solution, and 5 mL concentrated sulfuric acid were added in turn. After 30 min, the OD_485_ value was determined using UV-vis (Shimadzu UV3600), calculated as follows: soluble sugar content (mg g^−1^) = (108.49027 × OD_485_ − 18.99511)/[sample weight (g) × volume of extract solution for determination (mL)/volume of extract solution (mL)].

#### Chlorophyll content

The chlorophyll content was determined by the method described by Arnon.^69^ Leaves were placed in 95% ethanol solution and extracted in the dark until they had become completely discolored. After extraction, the absorption rates at 665 nm and 649 nm were measured with 95% ethanol as the control. Chlorophyll content was calculated as follows: chlorophyll *a* content (mg g^−1^) = (13.95 × OD_665_ − 6.88 × OD_649_) (mg L^−1^) × volume of extraction solution (mL) × dilution times/sample weight (g), chlorophyll *b* content (mg g^−1^) = (24.96 × OD_649_ − 7.32 × OD_665_) (mg L^−1^) × volume of extraction solution (mL) × dilution times/sample weight (g), chlorophyll content (mg g^−1^) = chlorophyll *a* content (mg g^−1^) + chlorophyll *b* content (mg g^−1^).

#### Malondialdehyde (MDA) content

The MDA content was determined using the thiobarbituric acid method.^70^ 0.5 g sample was placed into 2 mL phosphate buffer, then ground until homogenous before adding 5 mL 0.5% TBA solution and boiling for 10 min. Then, the mixture was centrifuged at 12 000 rpm for 15 min and the supernatant was aspirated after cooling. Finally, the absorption rates at 450 nm and 532 nm were measured, to determine the OD_450_ and OD_532_ values. MDA content was calculated as follows: MDA (mmol g^−1^ FW) = (6.452 × OD_532_ − 0.559 × OD_450_) × volume of extraction solution (mL)/(volume of extraction solution for determination (mL) × sample weight (g)).

#### Relative conductivity

The samples were soaked in distilled water for 2 h at 25 °C and the initial conductivity of the solution (*R*1) was determined. Afterwards, the mixture was heated for 30 min at 100 °C and the conductivity of the solution (*R*2) was determined after cooling. Calculation formula: relative conductivity (%) = *R*1/*R*2 × 100%.^71^

#### *F*_v_/*F*_m_

After an adequate dark adaptation period, the FluorCam Enclosed Chlorophyll Fluorescence Imaging System was used to measure the *F*_v_/*F*_m_ value.

### Statistical analysis

All treatments had at least three biological replicates and all measurements had at least three technical replicates. Values were expressed as means ± standard deviation (SD). One-way ANOVA was used for the statistical analysis and groups were compared with the Tukey or LSD test in DPS7.05. Different letters were used to represent significant differences (*P* < 0.05). The *F*-test ANOVA of all data was in Tables S3–S9.†

## Results and discussions

### Characterization of N-doped CDs

**Fig. 1:**
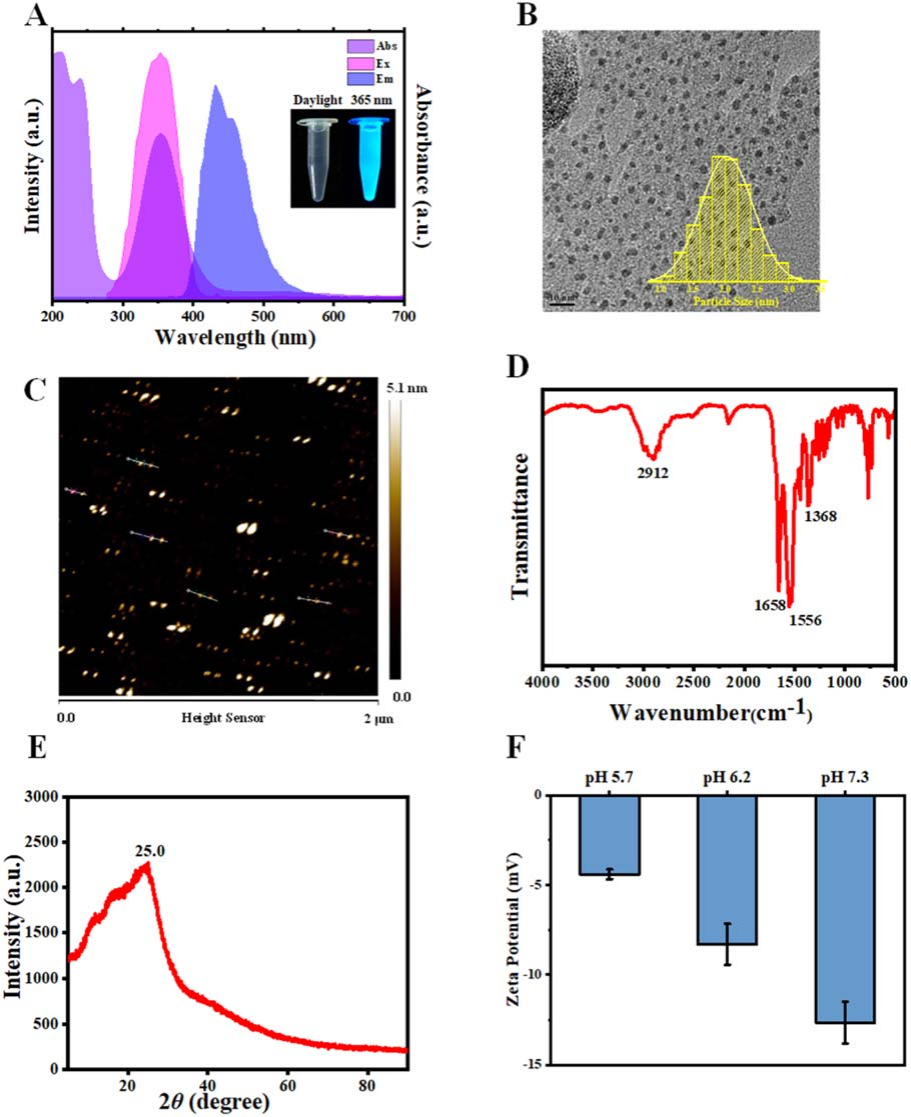
Chemical characterization of N-CDs. (A) UV-vis absorption and excitation and emission spectra of N-CDs. (B) Transmission electron microscopy (TEM) images of N-CDs and the size distribution of N-CDs (inserted image). (C) AFM image of N-CDs. (D) Fourier transform infrared (FTIR) spectra of N-CDs. (E) X-Ray diffraction (XRD) of N-CDs. (F) Zeta potential of N-CDs under pH 5.7/6.2/7.3 conditions. Error bars denote standard deviation.

N-CDs with UV-A light excitation and blue fluorescence emission were synthesized *via* the hydrothermal treatment of diethylenetriamine and citric acid at 180 °C for 4 h. As depicted in Fig. 1A, with 365 nm light irradiation, the N-CD solutions emitted blue fluorescence at 432 nm. Additionally, a strong absorption peak was observed at 350 nm, which was ascribed to the n–π* electronic transitions of the C=O bond.^52,54^ After synthesis, the N-CDs were morphologically characterized using TEM and AFM. As shown in Fig. 1B, C and S2A,† N-CDs exhibited spherical morphologies with an average particle diameter of 2.03 nm (TEM result) and height of 3.88 nm (AFM result). These results established the intrinsic nano-size features of N-CDs, which enable them to enter plant cells. The differences between the TEM and AFM results were likely due to the distinct measurement principles on which the two tests are based.^72,73^ The XRD profile of N-CDs exhibited a broad diffraction peak around 2*θ* of 25.0° (Fig. 1E), suggesting disordered carbon species. Subsequently, the chemical structure of the N-CDs was assessed using the FTIR and XPS spectra. Fig. 1D showed the FTIR spectrum of N-CDs. The peak at 1556 cm^−1^ belonged to the stretching vibration of C=C/C=N bonds. The strong vibration peak of carboxylate ions was observed around 1658 cm^−1^. The XPS analysis (Fig. S2B–E†) indicated that N-CDs were composed of C, N, and O elements with atomic ratios of 50.66%, 15.68%, and 27.68%, respectively. The high-resolution C 1s XPS spectrum illustrated that the peaks at 284.8, 286.4, 288.0, and 288.9 eV were attributed to the C–C/C=C, C–O/C–N, C=O/C=N, and O–C=O moieties, respectively. The O–C=O peak at 288.9 eV indicated the presence of carboxyl moieties on the surfaces of N-CDs. The *ξ*-potential characterization demonstrated that N-CDs presented negatively charged surfaces at pH 5.7–7.3 (Fig. 1F), suggesting there were carboxylic acid moieties on the N-CDs surfaces, which was in agreement with the XPS results. Furthermore, the corresponding optical band gap (*E*_g_) of N-CDs was estimated from the *Tauc* plot. Fig. S3† demonstrated a direct band gap (2.73 eV) of N-CDs, which was higher than that of undoped CDs (2.53 eV). The increase of *E*_g_ value induced by N-doping, decreases the recombination rate of electron–hole pairs, and probably facilitates the charge delocalization and electron transfer,^66,74,75^ which is probably favourable for N-CD-involved plant phenotype.^53^

### Absorption and distribution of N-CDs

**Fig. 2:**
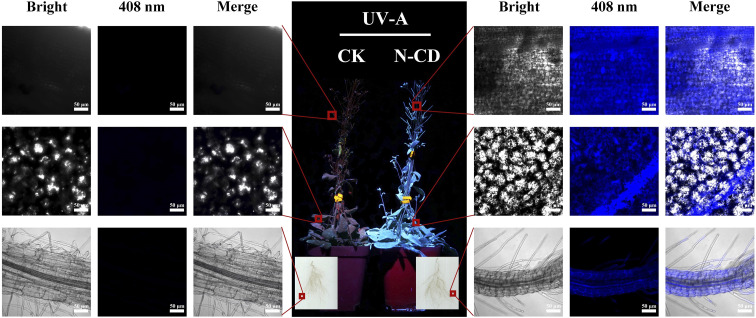
Absorption and distribution of N-CDs in *Arabidopsis*. (Middle) Picture captured under excitation light irradiation (365 nm). (Left and right) Confocal microscopy images of root, leaf, and fruit pods in seedlings treated with or without 300 mg L^−1^ N-CDs (408 nm excitation, 430–500 nm collection).

The uptake and transport of N-CDs in plants are curial for the N-CD-involved regulation of plant growth and development. The fluorescence properties of N-CDs can be employed to track their transport and distribution inside seedlings. After root irrigation with N-CDs (300 mg L^−1^) for 2 weeks, entire seedlings were rendered obviously blue due to the fluorescence of N-CDs, while the control seedlings exhibited red color, ascribed to the chloroplast autofluorescence (Fig. 2). Confocal microscopy (Fig. 2) showed that N-CDs were successively present in roots, leaves, and fruit pods. This indicated that the N-CDs could be absorbed by roots and transported to the above-ground tissues, such as leaves and fruit pods, a process likely driven primarily by transpiration. Furthermore, N-CDs could be observed in cells (Fig. 2). These results suggested that N-CDs can enter into cells, endowing themselves with the potential to regulate plant physiological activities, such as growth and stress resistance.

### N-CDs enhanced seedling growth *via* excitation light dependence

**Fig. 3:**
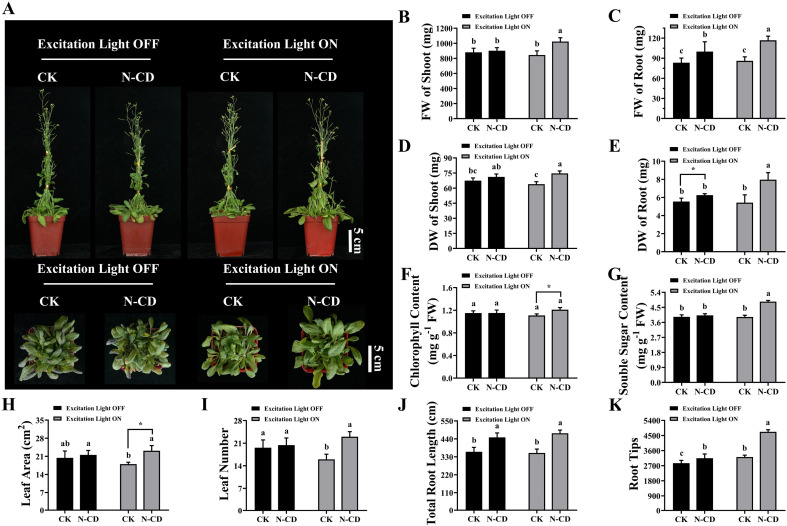
Effects of N-CDs on growth in the presence or absence of excitation light. (A) Effects of N-CDs (300 mg L^−1^) on the mature *Arabidopsis* seedlings on growth under light illumination with or without excitation light. Pictures captured after 20 days of cultivation. In the lower images in (A), the bolting stems of seedlings were cut to observe the seedling growth smoothly. (B–K) Fresh weights of shoots (B), fresh weights of roots (C), dry weights of shoots (D), dry weights of roots (E), chlorophyll contents (F), soluble sugar contents (G), leaf areas (H), leaf numbers (I), total root lengths (J), and root tips (K) in all treatments. All data (*n* ≥ 3) were analyzed using one-way analysis of variance (ANOVA), where different letters represent significant differences (*P* < 0.05, Tukey test).

After examining absorption, the effects of N-CDs on seedling growth were explored using *Arabidopsis* seedlings with a focus on the excitation light dependence. After root irrigation with N-CDs (300 mg L^−1^) for 20 days in the presence or absence of excitation light (365 nm), the growth phenotypes and associated indicators were measured (Fig. 3). Interestingly, under excitation light-free conditions, the N-CD-treated seedlings exhibited little difference in growth phenotype, fresh/dry weight of shoot, chlorophyll content, soluble sugar, and leaf area/number compared to the control group (Fig. 3). However, the underground tissues under N-CDs treatment increased (Fig. S4†), with increased total root length (24.51%) and root tip number (10.24%), thereby leading to increased fresh and dry weights of roots (19.86% and 12.70%, respectively). This phenomenon was likely due to the effects of CDs on endogenous auxin levels.^76,77^ Collectively, the N-CD-induced growth enhancement was relatively low and not significant in the absence of excitation light.

Meanwhile, several seedlings were treated with N-CD solutions (300 mg L^−1^) root irrigation in the presence of excitation light for 20 days (Fig. 3 and S4†). As expected, N-CD-treated seedlings showed significant increases in growth phenotypes, fresh and dry weights of shoots (21.07% and 16.87%, respectively), fresh and dry weights of roots (35.64% and 46.99%, respectively), chlorophyll content (9.17%), soluble sugar (23.41%), leaf area (28.68%), leaf number (43.75%), total root length (34.07%), and root tip numbers (46.69%) (Fig. 3B–I). What's more, as shown in Fig. S5,† the N-CD-treated seedling growth rate increased much more quickly in the presence of excitation light than those in the absence of excitation light. Notably, N-CD-treated seedlings presented outstanding increases in total root length (34.07%) and root tip number (46.69%) under excitation light irradiation, which were much greater than under excitation light-free conditions (Fig. 3J, K and S4†). To further confirm the influence of N-CDs on plant growth, seedlings of the woody plant *M. hupehensis* (Rosaceae family) were also treated with the N-CD solutions in the presence of excitation light. As in *Arabidopsis*, *M. hupehensis* exhibited significant growth enhancements in fresh weight, chlorophyll content, soluble sugar, stem length, leaf area, root length, and root surface area (Fig. S6†). Moreover, N-CDs also effectively promoted the absorption of nutrients, including the macro elements P, K, and Mg and the micro elements Fe, Zn, Cu, Mn, Mo, and B, which probably contributed to the enhanced seedling growth (Fig. S7†).

Based on these results, it was found that the N-CDs exerted a more pronounced enhancement effect on seedling growth in the presence of excitation light. As a proposed mechanism by which nanomaterials enhance photosynthesis, their optical properties are probably the main contributors to growth enhancement, facilitating electron/energy transfer^40,57,58^ and enhancing light conversion capacity.^59,60^ As shown in Fig. S8,† in these proposed mechanisms, when N-CDs are irradiated by excitation light, photogenerated electrons and holes are produced. The electrons can be transferred to chloroplasts where they enhance photosynthesis. The recombination irradiation of photogenerated electrons and holes results in fluorescence, converting excitation light to blue/red wavelengths that the photosynthesis system can employ. Therefore, N-CD-induced growth enhancement is excitation light-dependent to some extent. Furthermore, it has been reported that CDs can affect root development by influencing root apical auxin levels in illumination incubators,^76,77^ although the illumination light corresponding to these observations was not clearly characterized. This phenotypic effect was also observed in our work, with enhanced total root length and increased root tip number under excitation light.

### N-CDs induced salt tolerance without excitation light dependence

Having demonstrated the N-CD-induced growth enhancement, we subsequently explored the role of N-CDs on salt tolerance, focusing on the effect of excitation light. With the growing threat of climate change, plants are exposed to various adverse stresses at greater frequencies.^5^ In this work, seedlings were cultivated in 1/2 MS medium containing 0/50/100/150 mM NaCl in the presence and absence of excitation light (Fig. S9 and S10†). Inspiringly, in the presence of excitation light, N-CD-treated seedlings presented greater growth performance (Fig. S9†) in all NaCl-containing groups compared to the control. These N-CD-treated seedlings also exhibited greater fresh weights (16.25%, 27.06%, and 49.86%, respectively), chlorophyll content (7.82%, 11.75%, and 39.75%, respectively), and lower relative conductivity (12.35%, 11.79%, and 15.02%, respectively) and malondialdehyde (MDA) content (34.24%, 41.10%, and 39.76%, respectively) (Fig. S9†). Identical experiments were performed in the absence of excitation light, and contrary to the poor effect of N-CDs on enhancing growth under excitation light-free conditions, N-CDs effectively alleviated the salt stress without excitation light. Without excitation light, there were also significant increases in fresh weight (12.84%, 27.55%, and 33.47%, respectively) and chlorophyll content (14.56%, 14.20%, and 27.41%, respectively), and decreases in relative conductivity (14.44%, 12.60%, and 13.46%, respectively) and MDA content (20.11%, 23.08%, and 25.95%, respectively) relative to the control (Fig. S10†). It is worth noting that with no salt stress (0 mM NaCl), the N-CD-induced enhanced growth was also found to be excitation light-dependent in this experiment (Fig. S9 and S10†), which was in agreement with the results in Fig. 3.

**Fig. 4:**
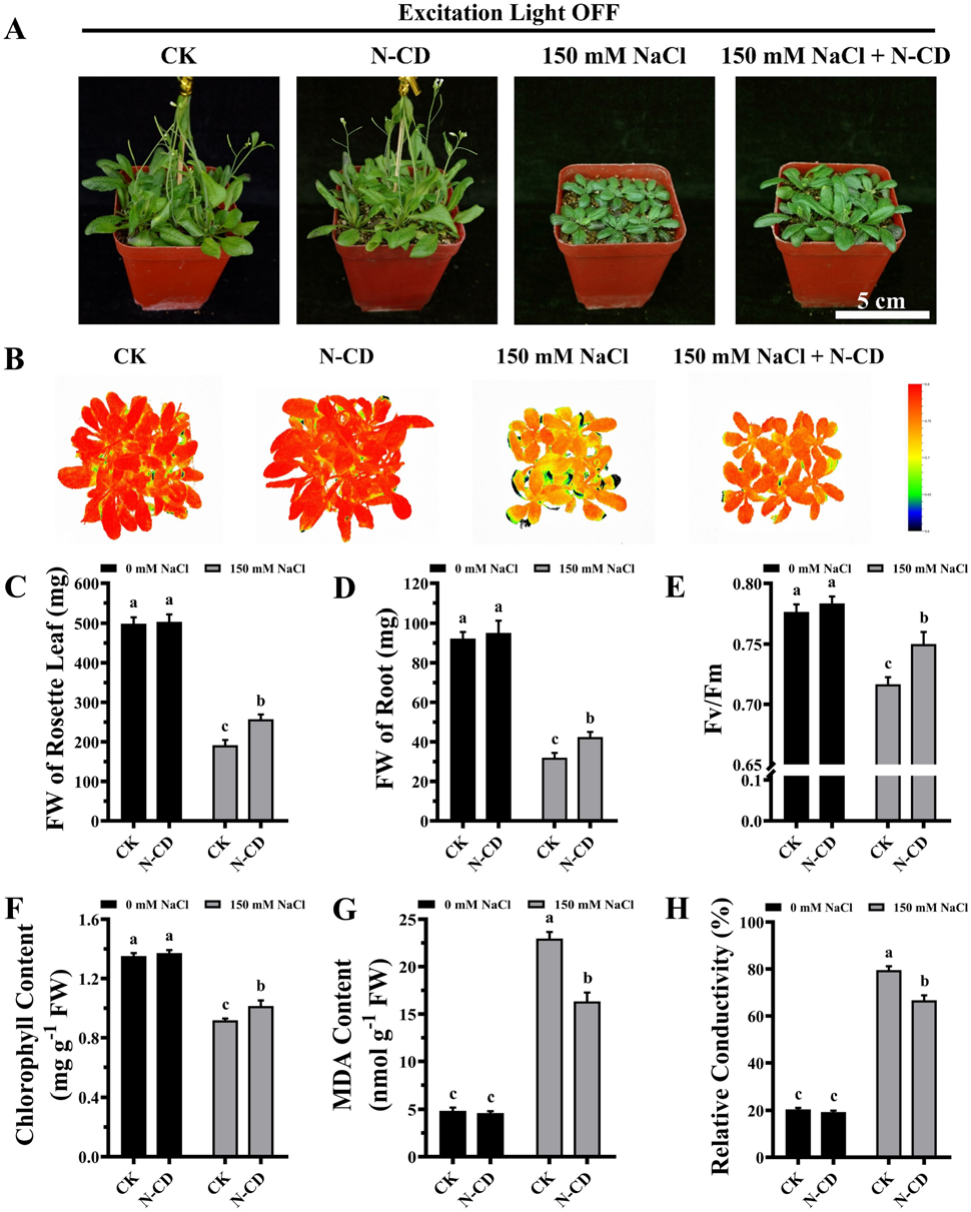
Effects of N-CDs in alleviating salt stress in the absence of excitation light. (A) Effects of N-CDs (300 mg L^−1^) on mature *Arabidopsis* seedlings in the mitigation of salt stress under light illumination without excitation light. Pictures captured after 14 days of cultivation. (B) Chlorophyll fluorescence images of all treatments. (C–H) Fresh weights of rosette leaves (C), fresh weights of roots (D), *F*_v_/*F*_m_ values (E), chlorophyll contents (F), MDA contents (G), and relative conductivities (H) of all treatments. All data (*n* ≥ 3) were analyzed using one-way ANOVA, where different letters represent significant differences (*P* < 0.05, Tukey test).

**Fig. 5:**
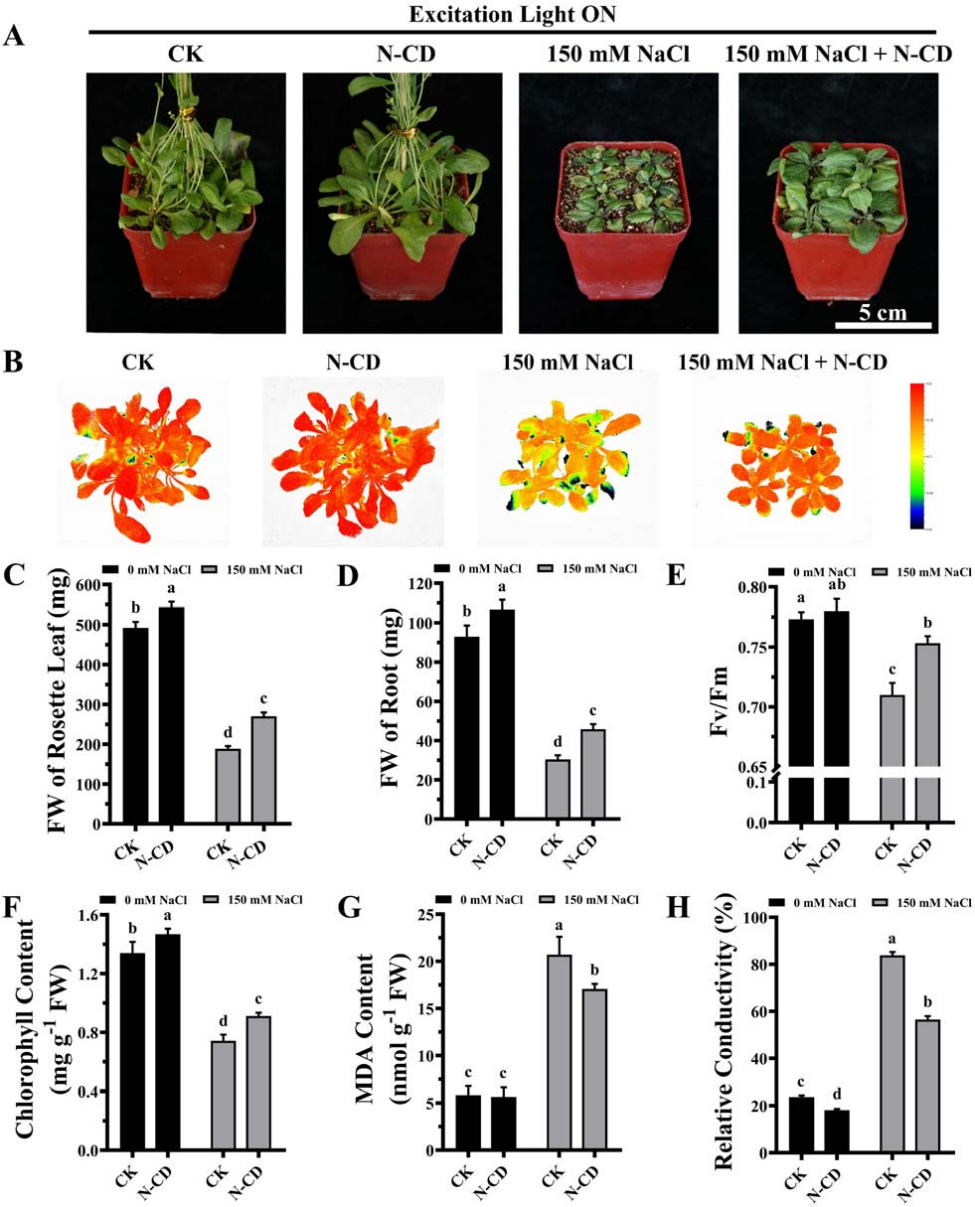
Effects of N-CDs in alleviating salt stress in the presence of excitation light. (A) Effects of N-CDs (300 mg L^−1^) on mature *Arabidopsis* seedlings in the mitigation of salt stress under light illumination with excitation light. Pictures captured after 14 days of cultivation. (B) Chlorophyll fluorescence images of all treatments. (C–H) Fresh weights of rosette leaves (C), fresh weights of roots (D), *F*_v_/*F*_m_ values (E), chlorophyll contents (F), MDA contents (G), and relative conductivities (H) of all treatments. All data (*n* ≥ 3) were analyzed using one-way ANOVA, where different letters represent significant differences (*P* < 0.05, Tukey test).

To further verify these results, mature *Arabidopsis* seedlings were irrigated with a 150 mM NaCl solution and cultivated in the presence or absence of excitation light. As shown in Fig. 4 and 5, N-CD-treated seedlings presented greater growth compared to the control groups under excitation light on/off conditions. With and without excitation light, the fresh weights of the rosette leaves increased by 43.29% and 34.22% and roots increased by 50.66% and 32.60%, respectively (Fig. 4C, D and 5C, D). Furthermore, chlorophyll contents increased by 22.59% and 10.45% with and without excitation light, respectively (Fig. 4F and 5F). Similar results were also found in the *F*_v_/*F*_m_ values (Fig. 4E and 5E), which reflect the health status of photosynthetic apparatus.^72^ The chlorophyll fluorescence images in Fig. 4B and 5B show that N-CD-treatment performed higher *F*_v_/*F*_m_ values, which indicated that N-CDs alleviated the damage to photosynthetic system caused by salt stress. Furthermore, the relative conductivity and MDA content, indirectly reflecting the extent of oxidative damage to the plant, exhibited significant decreases of 32.58% and 17.59%, and 16.13% and 28.84% when the excitation light was on and off, respectively (Fig. 4G, H and 5G, H). Collectively, these results suggested that N-CDs can effectively improve salt stress tolerance in an excitation light-independent manner. This improvement was slightly greater in the presence of excitation light, probably because N-CDs also enhanced seedling growth when exposed to excitation light.

Previous reports have indicated that the ROS scavenging capacity played a role in the CD-induced increase in stress tolerance.^49,62^ In this work, N-CDs enhanced salt tolerance independent of excitation light, suggesting that the ROS scavenging capacity of N-CDs is not related to their optical properties. In other words, the photo-generated electrons from light-excited N-CDs were not associated with their ROS scavenging capacity. Therefore, this ability probably originated from the intrinsic reduction capacity of N-CDs, such as the reductive raw materials of CDs. Recently, a mechanism for ROS-independent Ca^2+^ mobilization was proposed^48^ in which CDs served as a simultaneous Ca^2+^ signaling amplifier, with the hydroxyl and carboxyl groups on CDs contributing to this function. According to this mechanism, the optical properties of CDs should not be related to their Ca^2+^ mobilization ability and thereby stress tolerance role, in agreement with the results of this study. Taken together, N-CDs significantly enhance seedling growth and salt tolerance, but each role has different excitation light dependencies, indicating that N-CDs work *via* different pathways to achieve the two effects.

## Conclusions

In this work, N-CDs prepared using the hydrothermal method had strong enhancing effects on seedling growth and salt stress tolerance. Furthermore, the N-CD-induced growth enhancement was found to have a dependence on excitation light, while the N-CD-improved seedling salt tolerance did not require excitation light. This work provides new insight into plant nanoscience, which will pave the way for further agricultural applications of nanomaterials.

## Author contributions

The manuscript was designed and written through contributions of all authors. All authors have given approval to the final version of the manuscript.

## Conflicts of interest

There are no conflicts to declare.

## Supplementary Material

RA-013-D3RA01514A-s001
