# Supplementary material for: Nitrogen-doped carbon dots enhanced seedling growth and salt tolerance with distinct requirements of excitation light
Source: RSC Adv. 2023 Apr 18;13(18):12114–22. doi: 10.1039/d3ra01514a (PMC10111579; doi:10.1039/d3ra01514a)
Supplement: RA-013-D3RA01514A-s001 [file RA-013-D3RA01514A-s001.pdf]

Electronic Supplementary Material (ESI) for RSC Advances.
This journal is © The Royal Society of Chemistry 2023

Electronic Supplementary Information (ESI)

Nitrogen-doped carbon dots enhanced seedling growth and salt tolerance with distinct requirements of excitation light

Xiuli Jing,[a] Yankai Liu,[a] Xuzhe Liu,[b] Xiao-Fei Wang,[a] Chunxiang You,[a] Dayong Chang,[*c] and Shuai Zhang[*b]

[a] State Key Laboratory of Crop Biology, Shandong Green Fertilizer Technology Innovation Center, Apple technology innovation center of Shandong Province, College of Horticulture Science and Engineering, Shandong Agricultural University, Taian, Shandong, 271018, China.

[b] Key Laboratory of Agricultural Film Application of Ministry of Agriculture and Rural Affairs, College of Chemistry and Material Science, Shandong Agricultural University, Taian, Shandong, 271018, China.

[c] Yantai Goodly Biological Technology Co., Ltd, Yantai, Shandong, China.

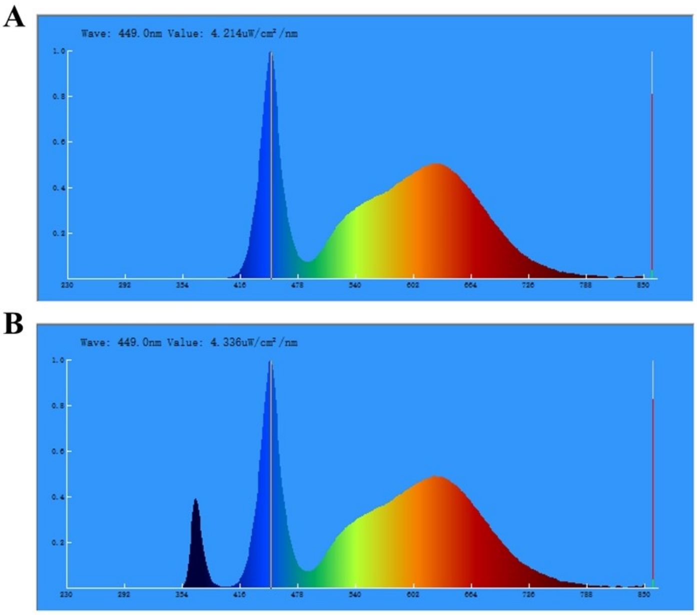

Fig. S1 (A) Seedlings growth light spectrum without UV-A light. (B) Seedlings growth light spectrum containing UV-A light (λ_{max} = 365 nm).

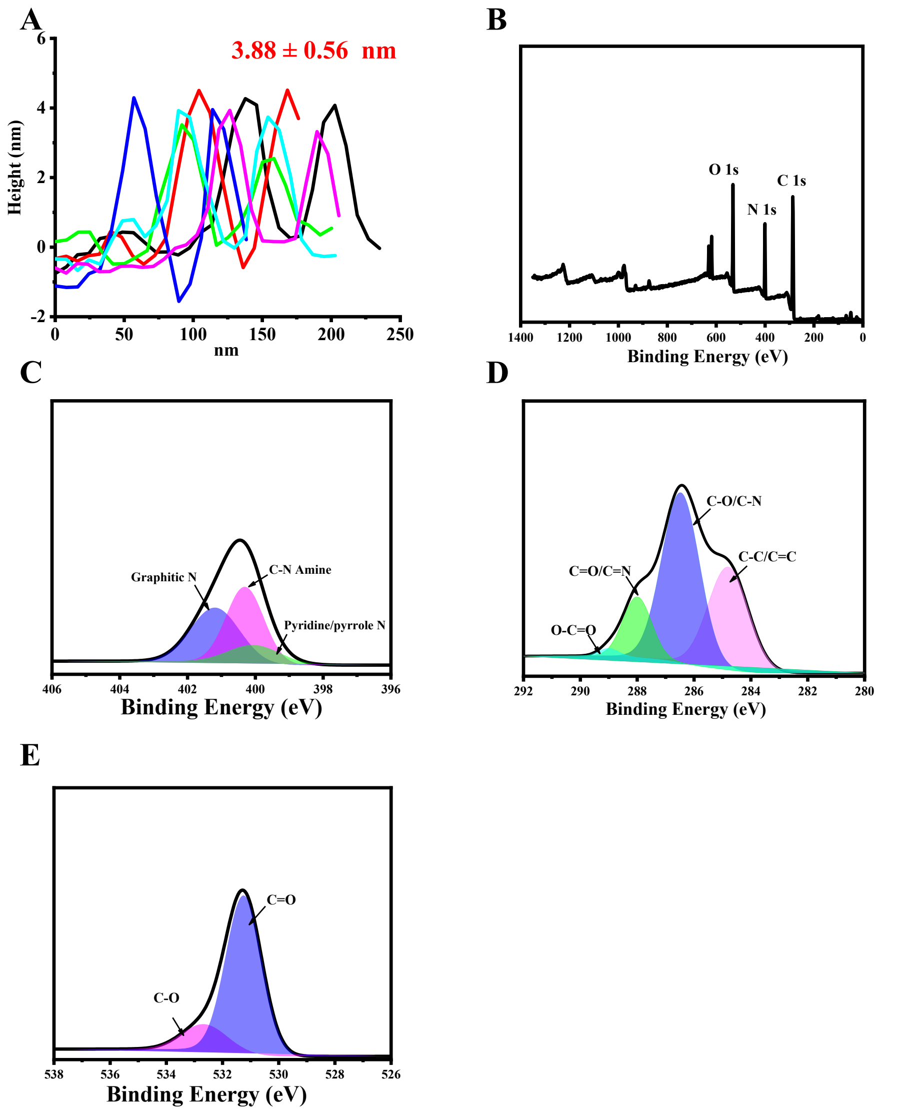

Fig. S2 (A)The height of N-CD. (B) XPS full scan spectrum of N-CD. (C-E) High-resolution XPS spectra of N_{1s} (C), C_{1s} (D), O_{1s} (E) of N-CD.

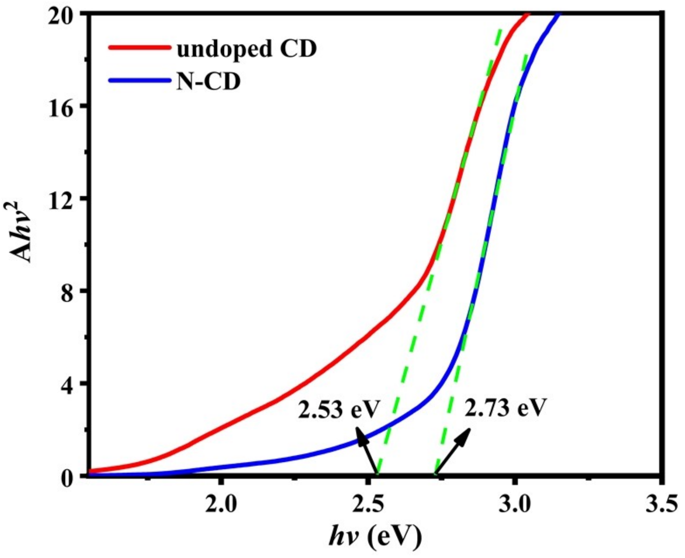

Fig. S3 The optical band gap of the N-CD and undoped CD.

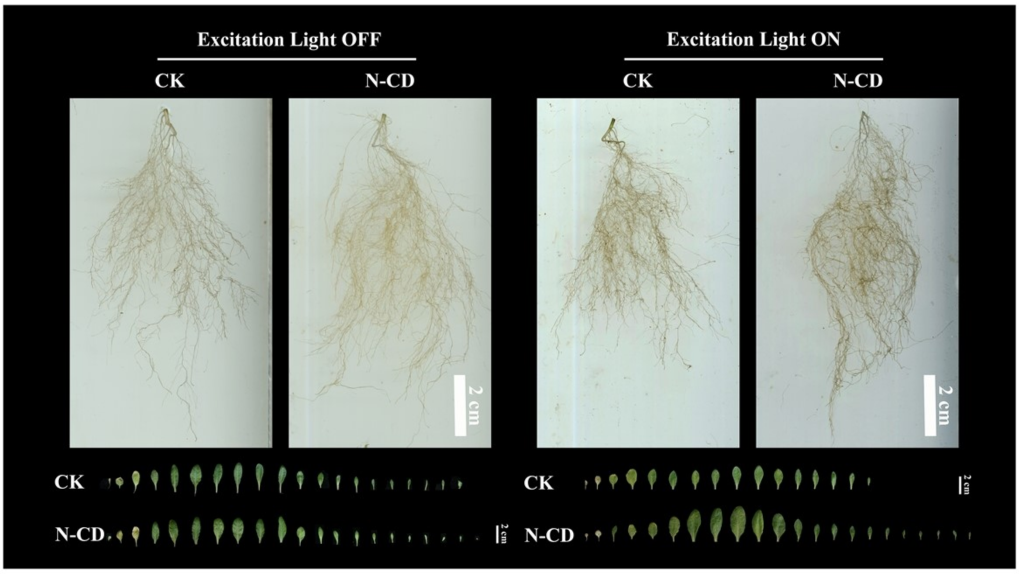

Fig. S4 Effects of N-CDs on root development and leaf growth for 20 days treatment in *Arabidopsis*.

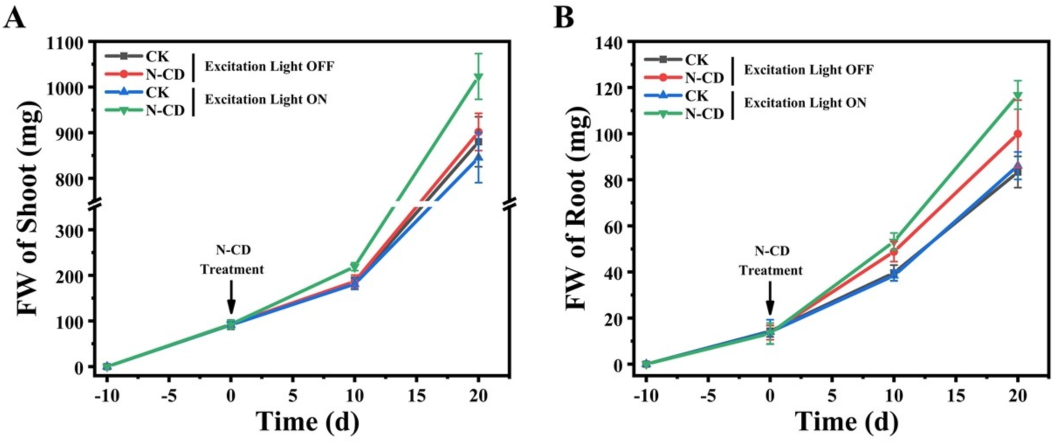

Fig. S5 The fresh weights of shoot (A) and root (B) of N-CD-treated seedlings at different time with or without excitation light.

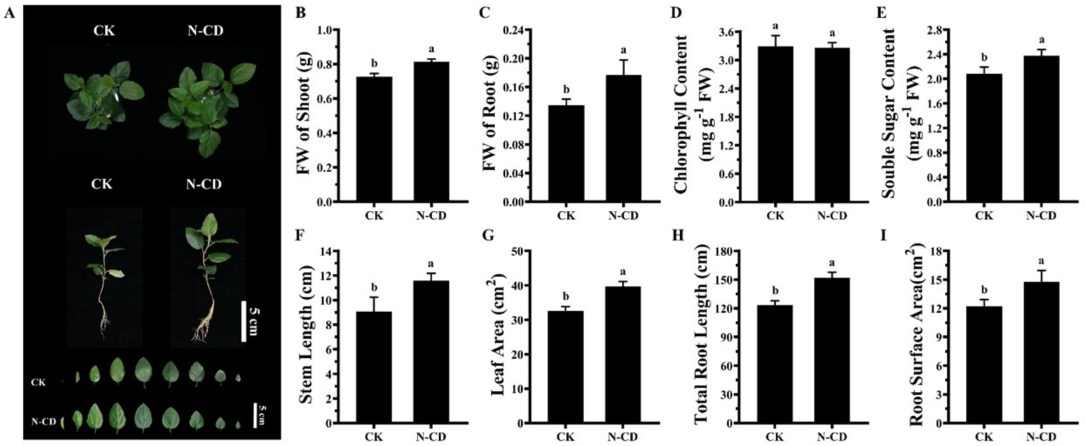

Fig. S6 Effects of N-CDs on improving the growth of *Malus Hupehensis* in the presence of excitation light. (A) Effects of N-CDs (300 mg L^{-1}) on the *M. Hupehensis* seedlings on the enhancement of under light illumination (20.4267 μmol m^{-2} s^{-1}) with excitation light (λ_{max} = 365 nm, 0.8325 μmol m^{-2} s^{-1}). Pictures captured after 30 days of cultivation. (B-I) Fresh weight of shoot (B), fresh weight of root (C), chlorophyll content (D), soluble sugar content (E), stem length (F), leaf area (G), total root length (H), root surface area (I) of all treatments. All data (n ≥ 3) were analyzed by one-way ANOVA, the different letters represent significant differences ($P < 0.05$, Tukey test).

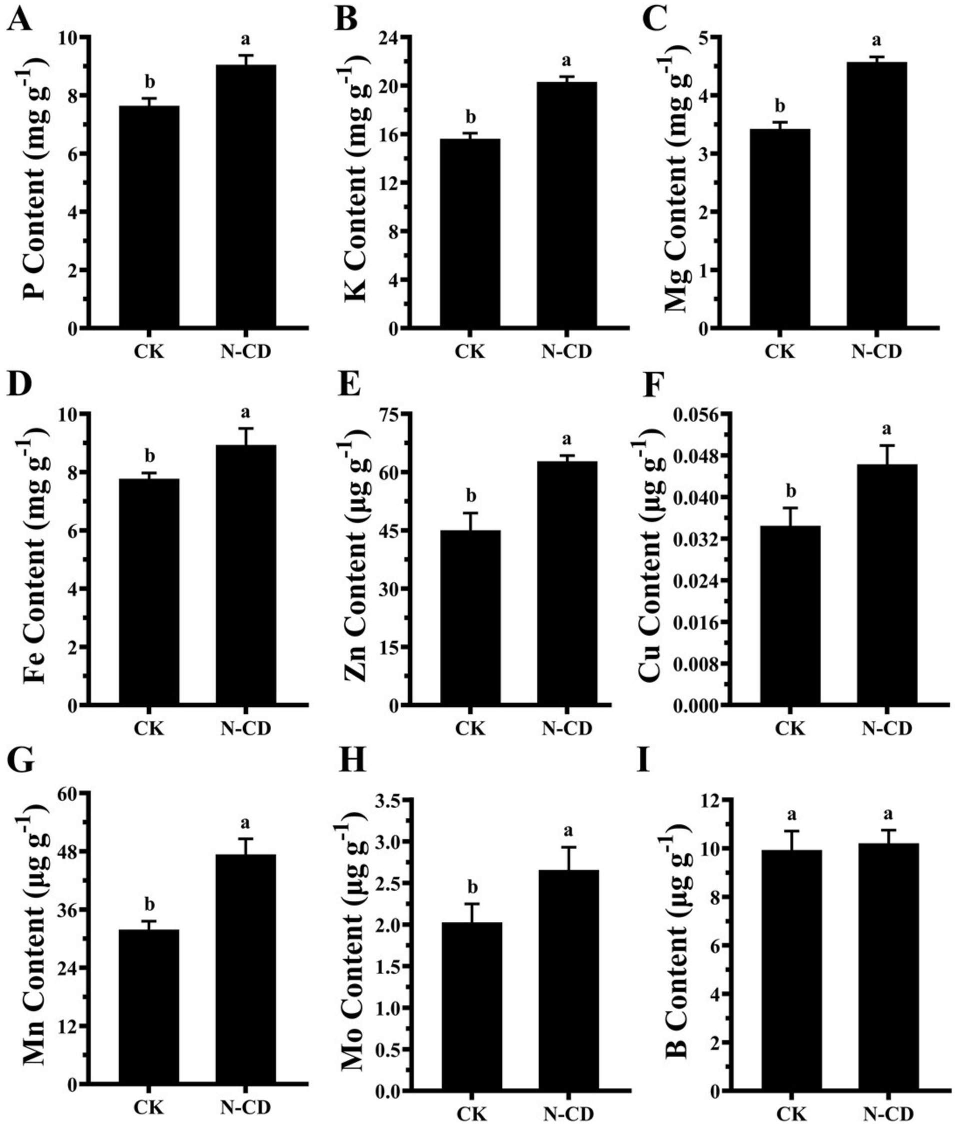

Fig. S7 Effects of N-CDs on promoting the absorption of nutrients of *M. Hupehensis* in the presence of excitation light. All data ($n \geq 3$) were analyzed by one-way ANOVA, the different letters represent significant differences ($P < 0.05$, Tukey test).

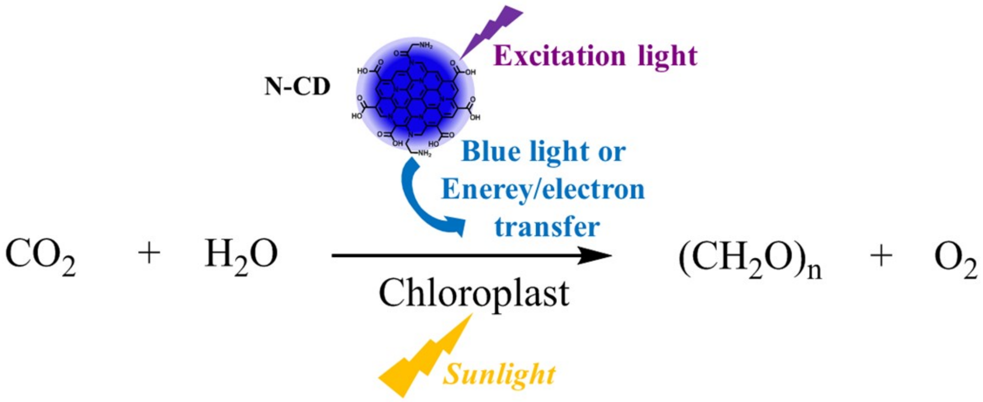

Fig. S8 The chemical formula of N-CD-induced plant growth and photosynthesis enhancement.

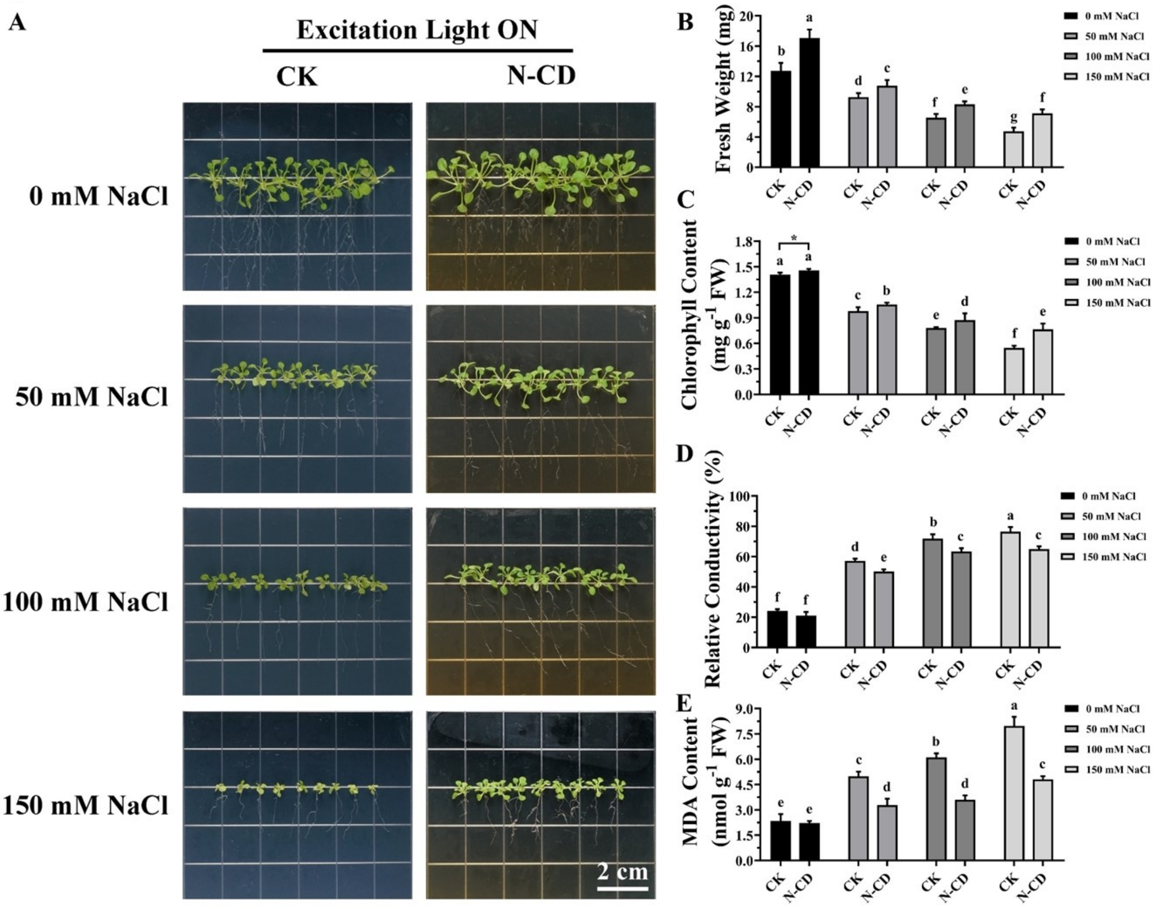

Fig. S9 Effects of N-CDs in alleviating salt stress in the presence of excitation light. (A) Effects of N-CDs (300 mg L^{-1}) on the *Arabidopsis* seedlings in the mitigation of salt stress under light illumination (20.4267 μmol m^{-2} s^{-1}) with excitation light (λ_{max} = 365 nm, 0.8325 μmol m^{-2} s^{-1}). Pictures captured after 14 days of cultivation. (B-E) Fresh weight (B), chlorophyll content (C), relative conductivity (D), MDA content (E) of all treatments. All data (n ≥ 3) were analyzed by one-way ANOVA, the different letters represent significant differences ($P < 0.05$, LSD test).

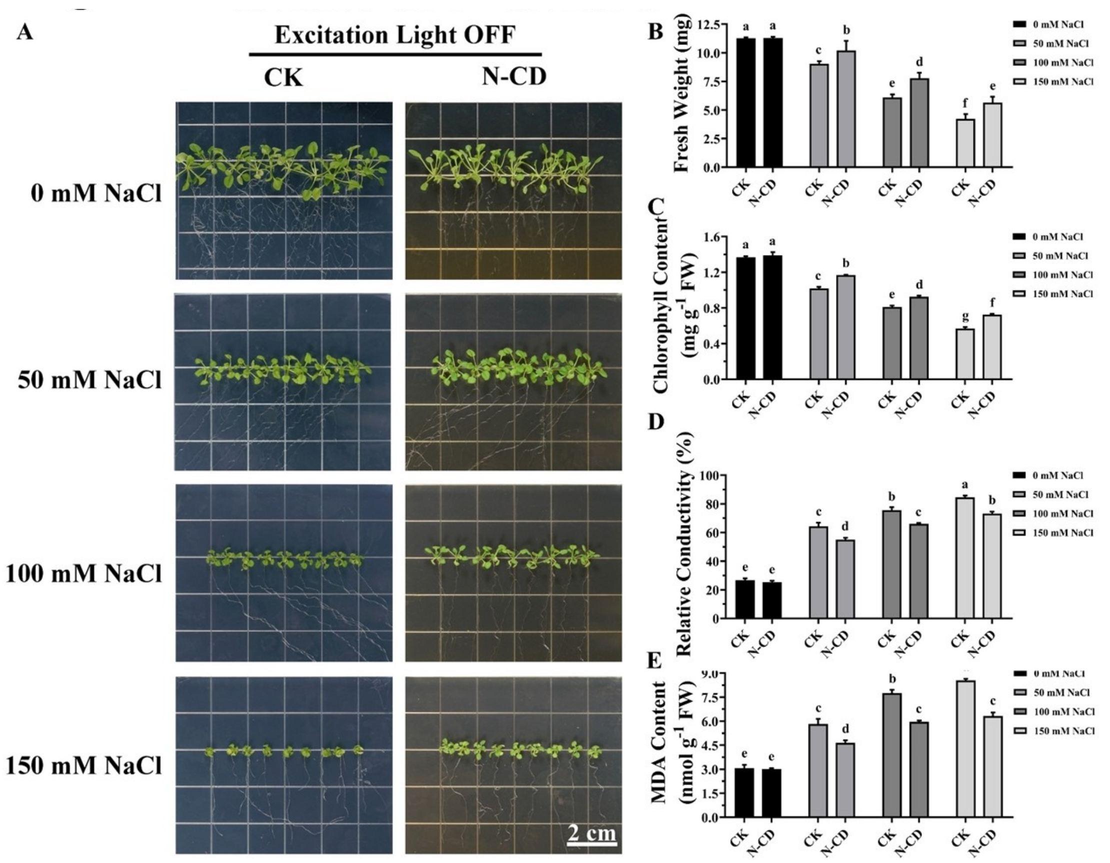

Fig. S10 Effects of N-CDs in alleviating salt stress in the absence of excitation light. (A) Effects of N-CDs (300 mg L^{-1}) on the *Arabidopsis* seedlings in the mitigation of salt stress under light illumination (20.4267 μmol m^{-2} s^{-1}). Pictures captured after 14 days of cultivation. (B-E) Fresh weight (B), chlorophyll content (C), relative conductivity (D), MDA content (E) of all treatments. All data (n ≥ 3) were analyzed by one-way ANOVA, the different letters represent significant differences ($P < 0.05$, LSD test).

Table S1. Hoagland nutrient solution.

	Nutritive Salt	Concentration (g L^{-1})	Amount of various stores per 100 mL culture solution (mL)
Macroelement	$Ca(NO_3)_2 \cdot 4H_2O$	236	0.5
	KNO_3	102	
	$MgSO_4 \cdot 7H_2O$	98	
	KH_2PO_4	27	
	EDTA-Na	7.45	
	$FeSO_4 \cdot 7H_2O$	5.57	
Microelement	H_3BO_4	2.86	0.1
	$MnCl_2 \cdot 4H_2O$	1.81	
	$CuSO_4 \cdot 5H_2O$	0.08	
	$ZnSO_4 \cdot 7H_2O$	0.22	
	$H_2MoO_4 \cdot H_2O$	0.09	

Table S2. Light intensity of different wavelengths of light in seedling growth

Parameters	Light conditions with excitation light (μmol m^{-2} s^{-1})	Light conditions without excitation light (μmol m^{-2} s^{-1})
PPFD	20.4267	20.3524
PPFD-UV	0.8325	0.0207
PPFD-B	4.9127	4.7881
PPFD-G	6.2135	6.2561
PPFD-R	9.30	9.31
PPFD-FR	1.63032	1.59390
PPFD-IR	0.20935	0.22377

Table S3. The F-test ANOVA of Fig. 3.

Analysis of Variance Table (Fig. 3)						
	**Soruces of variation**	**SS**	**df**	**MS**	**F**	**p-value**
**Fig. 3B FW of Shoot**	Between-processing	179656.7583	3	59885.5861	23.562	0.0001
	Within-processing	91497.2574	36	2541.5905		
	Total variation	271154.0157	39			
**Fig. 3C FW of Root**	Between-processing	7040.5168	3	2346.8389	28.099	0.0001
	Within-processing	3006.7167	36	83.5199		
	Total variation	10047.2335	39			
**Fig. 3D DW of Shoot**	Between-processing	325.4975	3	108.4992	16.13	0.0001
	Within-processing	107.6253	16	6.7266		
	Total variation	433.1228	19			
**Fig. 3E DW of Root**	Between-processing	20.5571	3	6.8524	18.167	0.0001
	Within-processing	6.0352	16	0.3772		
	Total variation	26.5923	19			
**Fig. 3F Chlorophyll Content**	Between-processing	0.0157	3	0.0052	3.285	0.0793
	Within-processing	0.0128	8	0.0016		
	Total variation	0.0285	11			
**Fig. 3G Soluble Sugar Content**	Between-processing	1.7807	3	0.5936	59.282	0.0001
	Within-processing	0.0801	8	0.01		
	Total variation	1.8608	11			
**Fig. 3H Leaf Area**	Between-processing	70.5508	3	23.5169	6.506	0.0044
	Within-processing	57.8312	16	3.6144		
	Total variation	128.382	19			
**Fig. 3I Leaf Number**	Between-processing	125.35	3	41.7833	10.714	0.0004
	Within-processing	62.4	16	3.9		
	Total variation	187.75	19			
**Fig. 3J Total Root Length**	Between-processing	112589.3877	3	37529.7959	62.432	0.0001
	Within-processing	21640.772	36	601.1326		
	Total variation	134230.1596	39			
**Fig. 3K Root Tips**	Between-processing	21191170.22	3	7063723.406	240.52	0.0001
	Within-processing	1057268.225	36	29368.5618		
	Total variation	22248438.44	39			

Table S4. The F-test ANOVA of Fig. 4.

Analysis of Variance Table (Fig. 4)						
	**Soruces of variation**	**SS**	**df**	**MS**	**F**	**p-value**
**Fig. 4C FW of Rosette Leaf**	Between-processing	394672.5604	3	131557.5201	563.067	0.0001
	Within-processing	3738.3104	16	233.6444		
	Total variation	398410.8708	19			
**Fig. 4D FW of Root**	Between-processing	16267.9897	3	5422.6632	350.837	0.0001
	Within-processing	247.302	16	15.4564		
	Total variation	16515.2917	19			
**Fig. 4E F_v/F_m**	Between-processing	0.0083	3	0.0028	55.111	0.0001
	Within-processing	0.0004	8	0.0001		
	Total variation	0.0087	11			
**Fig. 4F Chlorophyll Content**	Between-processing	0.4832	3	0.1611	270.11	0.0001
	Within-processing	0.0048	8	0.0006		
	Total variation	0.488	11			
**Fig. 4G MDA Content**	Between-processing	735.1777	3	245.0592	653.099	0.0001
	Within-processing	3.0018	8	0.3752		
	Total variation	738.1795	11			
**Fig. 4H Relative Conductivity**	Between-processing	8759.2454	3	2919.7485	1498.909	0.0001
	Within-processing	15.5833	8	1.9479		
	Total variation	8774.8287	11			

Table S5. The F-test ANOVA of Fig. 5.

Analysis of Variance Table (Fig. 5)						
	**Soruces of variation**	**SS**	**df**	**MS**	**F**	**p-value**
**Fig. 5C FW of Rosette Leaf**	Between-processing	439871.1375	3	146623.7125	1092.017	0.0001
	Within-processing	2148.3	16	134.2688		
	Total variation	442019.4375	19			
**Fig. 5D FW of Root**	Between-processing	20165.2	3	6721.7333	412.376	0.0001
	Within-processing	260.8	16	16.3		
	Total variation	20426	19			
**Fig. 5E Fv/Fm**	Between-processing	0.009	3	0.003	44.792	0.0001
	Within-processing	0.0005	8	0.0001		
	Total variation	0.0095	11			
**Fig. 5F Chlorophyll Content**	Between-processing	1.0672	3	0.3557	152.326	0.0001
	Within-processing	0.0187	8	0.0023		
	Total variation	1.0859	11			
**Fig. 5G MDA Content**	Between-processing	539.1376	3	179.7125	125.201	0.0001
	Within-processing	11.4831	8	1.4354		
	Total variation	550.6208	11			
**Fig. 5H Relative Conductivity**	Between-processing	8461.9476	3	2820.6492	2357.734	0.0001
	Within-processing	9.5707	8	1.1963		
	Total variation	8471.5184	11			

Table S6. The F-test ANOVA of Fig. S6.

Analysis of Variance Table (Fig. S6)						
	**Soruces of variation**	**SS**	**df**	**MS**	**F**	**p-value**
**Fig. S6B FW of Shoot**	Between-processing	0.011	1	0.011	41.514	0.003
	Within-processing	0.0011	4	0.0003		
	Total variation	0.0121	5			
**Fig. S6C FW of Root**	Between-processing	0.0027	1	0.0027	10.257	0.0328
	Within-processing	0.001	4	0.0003		
	Total variation	0.0037	5			
**Fig. S6D Chlorophyll Content**	Between-processing	0.0018	1	0.0018	0.06	0.8192
	Within-processing	0.1211	4	0.0303		
	Total variation	0.1229	5			
**Fig. S6E Soluble Sugar Content**	Between-processing	0.2144	1	0.2144	19.552	0.0022
	Within-processing	0.0877	8	0.011		
	Total variation	0.3021	9			
**Fig. S6F Stem Length**	Between-processing	15.6763	1	15.6763	18.198	0.0027
	Within-processing	6.8913	8	0.8614		
	Total variation	22.5676	9			
**Fig. S6G Leaf Area**	Between-processing	125.6029	1	125.603	79.072	0.0001
	Within-processing	12.7077	8	1.5885		
	Total variation	138.3106	9			
**Fig. S6H Total Root Length**	Between-processing	2025.5239	1	2025.52	83.907	0.0001
	Within-processing	193.1205	8	24.1401		
	Total variation	2218.6444	9			
**Fig. S6I Root Surface Area**	Between-processing	16.4771	1	16.4771	18.14	0.0028
	Within-processing	7.2664	8	0.9083		
	Total variation	23.7435	9			

Table S7. The F-test ANOVA of Fig. S7.

Analysis of Variance Table (Fig. S7)						
	**Soruces of variation**	**SS**	**df**	**MS**	**F**	**p-value**
**Fig. S7A P Content**	Between-processing	2.9816	1	2.9816	34.978	0.0041
	Within-processing	0.341	4	0.0852		
	Total variation	3.3226	5			
**Fig. S7B K Content**	Between-processing	33.0111	1	33.0111	165.439	0.0002
	Within-processing	0.7981	4	0.1995		
	Total variation	33.8092	5			
**Fig. S7C Mg Content**	Between-processing	1.9717	1	1.9717	192.754	0.0002
	Within-processing	0.0409	4	0.0102		
	Total variation	2.0126	5			
**Fig. S7D Fe Content**	Between-processing	2.0169	1	2.0169	11.249	0.0285
	Within-processing	0.7172	4	0.1793		
	Total variation	2.7341	5			
**Fig S7E Zn Content**	Between-processing	471.4483	1	471.4483	44.165	0.0027
	Within-processing	42.6993	4	10.6748		
	Total variation	514.1476	5			
**Fig. S7F Cu Content**	Between-processing	0.0002	1	0.0002	16.971	0.0146
	Within-processing	0	4	0		
	Total variation	0.0003	5			
**Fig. S7G Mn Content**	Between-processing	360.4139	1	360.4139	54.612	0.0018
	Within-processing	26.3981	4	6.5995		
	Total variation	386.812	5			
**Fig. S7H Mo Content**	Between-processing	0.5925	1	0.5925	9.62	0.0362
	Within-processing	0.2464	4	0.0616		
	Total variation	0.8389	5			
**Fig. S7I B Content**	Between-processing	0.116	1	0.116	0.255	0.6403
	Within-processing	1.8211	4	0.4553		
	Total variation	1.937	5			

Table S8. The F-test ANOVA of Fig. S9.

Analysis of Variance Table (Fig. S9)						
	**Soruces of variation**	**SS**	**df**	**MS**	**F**	**p-value**
**Fig. S9B Fresh Weight**	Between-processing	537.1804	7	76.7401	147.101	0.0001
	Within-processing	16.6938	32	0.5217		
	Total variation	553.8742	39			
**Fig. S9C Chlorophyll Content**	Between-processing	2.1022	7	0.3003	159.463	0.0001
	Within-processing	0.0301	16	0.0019		
	Total variation	2.1323	23			
**Fig. S9D Relative Conductivity**	Between-processing	9100.4014	7	1300.0573	291.151	0.0001
	Within-processing	71.4438	16	4.4652		
	Total variation	9171.8452	23			
**Fig. S9E MDA Content**	Between-processing	81.175	7	11.5964	108.721	0.0001
	Within-processing	1.7066	16	0.1067		
	Total variation	82.8816	23			

Table S9. The F-test ANOVA of Fig. S10.

Analysis of Variance Table (Fig. S10)						
	**Soruces of variation**	**SS**	**df**	**MS**	**F**	**p-value**
**Fig. S10B Fresh Weight**	Between-processing	252.5388	7	36.077	191.028	0.0001
	Within-processing	6.0434	32	0.1889		
	Total variation	258.5822	39			
**Fig. S10C Chlorophyll Content**	Between-processing	1.8501	7	0.2643	774.916	0.0001
	Within-processing	0.0055	16	0.0003		
	Total variation	1.8556	23			
**Fig. S10D Relative Conductivity**	Between-processing	10222.2473	7	1460.321	674.771	0.0001
	Within-processing	34.6268	16	2.1642		
	Total variation	10256.874	23			
**Fig. S10E MDA Content**	Between-processing	83.9114	7	11.9873	326.93	0.0001
	Within-processing	0.5867	16	0.0367		
	Total variation	84.4981	23			
